# Long-term changes in herbivore community and vegetation impact of wild and domestic herbivores across Iceland

**DOI:** 10.1007/s13280-024-01998-6

**Published:** 2024-02-25

**Authors:** Mathilde Defourneaux, Isabel C. Barrio, Noémie Boulanger-Lapointe, James D. M. Speed

**Affiliations:** 1 Faculty of Environmental and Forest Sciences, Agricultural University of Iceland, Árleyni 22, Keldnaholt, 112 Reykjavík, Iceland; 2 Department of Geography, University of Victoria, Victoria, BC Canada; 3 Department of Natural History, NTNU University Museum, Norwegian University of Science and Technology, 7491 Trondheim, Norway

**Keywords:** Herbivory, Land management, Livestock, Metabolic biomass, Vegetation impact, Wildlife conflicts

## Abstract

Changes in wild and domestic herbivore populations significantly impact extensive grazing systems, particularly in low productive environments, where increasing wild herbivore populations are perceived as a threat to farming. To assess the magnitude of these changes in Iceland, we compiled time series on herbivore populations from 1986 to 2020 and estimated changes in species densities, metabolic biomass, and consumption of plant biomass in improved lands and unimproved rangelands. We compared estimates of consumption rates to past and present net primary production. Overall, the herbivore community composition shifted from livestock to wildlife dominated. However, wild herbivores only contributed a small fraction (14%) of the total herbivore metabolic biomass and consumption (4–7%), and livestock dominated the overall herbivore biomass. These insights highlight the necessity of developing improved local integrated management for both wild and domestic herbivores where they coexist.

**Supplementary Information:**

The online version contains supplementary material available at 10.1007/s13280-024-01998-6.

## Introduction

By consuming plants, depositing waste and trampling, herbivores have a strong effect on ecosystem processes and functions (Olofsson 2009; Forbes et al. 2019). However, the magnitude and direction of herbivore impacts depend on multiple environmental factors, including ecosystem productivity (Burkepile and Parker 2017), and herbivore densities (Austrheim et al. 2014) and identities (Hempson et al. 2015; Wang et al. 2019). For instance, African savannas experience vegetation shifts with declines in grass or shrubs depending on the balance between grazing and browsing herbivores (Staver et al. 2021). Thus, changes in the composition of herbivore communities can determine the impact that herbivores have on ecosystems.

Worldwide, human management and environmental changes are driving changes in herbivore populations. The escalating demand for meat has led to increased livestock production in most countries, often replacing wild species and homogenizing herbivore communities (Hempson et al. 2017). Most of this intensification has occurred in managed agricultural lands, while rangelands have been abandoned in regions with high intensification of meat and dairy production (Alkemade et al. 2013; Godde et al. 2018). Conversely, wild species can benefit from changes in agricultural practices; for example, the abundance of migratory waterfowl has increased in the Arctic because of the enhanced food availability in their wintering ranges (Fox and Abraham 2017), and the densities of wild cervids have increased with recent changes in land use and population management in Norway, effectively replacing grazing livestock in some regions (Speed et al. 2019). The interplay between wild and domestic herbivores poses both management and societal challenges, particularly in low-productive ecosystems where increasing herbivore populations may constrain primary productivity and exacerbate conflicts between stakeholders (Mysterud and Austrheim 2008). Indeed, the spatial overlap between livestock and wild herbivores is often interpreted as a potential indicator of resource competition and is perceived as a threat to farming industries, while also potentially affecting populations of protected wildlife (Pozo et al. 2021). Understanding the relative impact of domestic and wild species is crucial for effective management policies in a changing world.

In Iceland, the vertebrate herbivore community is composed of a mixture of wild and domestic animals, where birds are the only native herbivores. In the last decades, changes in agricultural policies have led to a reduction in the abundance of sheep by nearly half, following the introduction of a livestock quota in the early 1980s (Arnalds and Barkarson 2003). In turn, as in other parts of the Arctic, the numbers of migratory waterfowl (i.e., Anatidae) have greatly increased (Smith et al. 2020). Upon arrival in Iceland in early spring, geese and swans stage in improved agricultural lands (Fox et al. 1992), where livestock also graze for part of the season. The potential competition for resources between livestock and wild herbivores on improved grasslands (i.e., fertilized pastures including hay fields and grazing pastures) is becoming a concern for farmers (Jóhannesdóttir et al. 2017). Such concerns are extending to less productive rangelands in the highlands, where sheep graze during the summer months and spatially overlap with waterfowl suggesting that competition can also occur (Boulanger-Lapointe et al. 2022). However, limited evidence exists on the impact of waterfowl on sheep farming, and the broader consequences of herbivore community changes in both improved and unimproved land remain unclear.

This study aims to assess changes in the Icelandic herbivore community over the last decades and their impact on vegetation at a national scale. Specifically, our questions are as follows: (1) has herbivore community composition shifted over time? We anticipate a transition from a livestock-dominated to a wild-dominated system due to changes in Icelandic agricultural policies and an increasing migratory goose population. If such a shift has occurred, (2) do changes in herbivore community composition correlate with shifts in densities of particular herbivore species? And (3) are these changes reflected more broadly in the relative metabolic biomass (i.e., the animals’ daily energy requirements) of wild and domestic herbivores? While the overall herbivore population may still grow, community shifts are likely related to fewer sheep and more waterfowl, with increasing metabolic biomass of wild herbivores relative to domestic species. Given their differing sizes and energy needs, we wondered: (4) do these changes translate to divergent impacts of wild and domestic herbivores on net primary production of improved and unimproved lands?.

## Materials and methods

### Study area

This study focuses on grazing areas used by domestic and wild herbivores in Iceland, which encompass 60% of the country (Stefánsson et al. 2020). About 40% of the grazing areas consist of fenced, improved grasslands located close to the farms in the lowlands (i.e., below 200 m a.s.l.). Improved grasslands are privately owned by farmers and are managed through fertilization and planting. These improved lands are used for hay production and/or as grazing pastures. In contrast, unimproved rangelands are mainly communally owned lands usually located at higher elevations characterized by low-productivity subarctic tundra vegetation, and used for extensive livestock grazing. Rangelands are managed as natural ecosystems and include a mosaic of tundra habitats, including heathlands, wetlands and natural grasslands (Thorhallsdottir 1997).

### Vertebrate herbivore community

Iceland’s vertebrate herbivore community encompasses domestic and wild species, including a total of 11 vertebrate herbivores (Table 1). Introduced by the first settlers in the ninth century, domestic livestock include sheep (*Ovis aries)*, cattle (*Bos taurus*), horses (*Equus ferus caballus*) and goats (*Capra hircus*) (Thomson and Simpson 2006). All livestock receive supplementary food during winter when, except for horses, animals are kept indoors. Cattle, goats and sheep are released to improved pastures in spring (Fridriksson 1972). Sheep are then grazed on unimproved rangelands from mid-June to mid-September (Ross et al. 2016). Horses mainly use unimproved rangelands year-round, but can be moved to improved pastures during the mating season (Magnússon and Magnusson 1990). On unimproved rangelands, horses use a more restricted area than sheep (unmapped), usually at lower elevations and closer to farms (i.e., horses do not graze in most highland rangelands).

**Table 1:** The vertebrate herbivore community in Iceland includes domestic and wild species that are native or introduced. Their use of improved and unimproved grazing areas in Iceland is briefly described

Species	Status	Use of grazing areas in Iceland	
		Improved lands	Unimproved lands
Cattle (*Bos taurus* L.)	Domestic, introduced	Grazing pastures during growing season (May to Sept)	-
Icelandic Horse (*Equus ferus caballus* L.)	Domestic, introduced	–	Mostly grazed on rangelands year-round, but in the last 30 years, lowland fens have increased in importance as grazing area during summer
Icelandic sheep (*Ovis aries* L.)	Domestic, introduced	Grazing pastures during spring and autumn, i.e., after lambs are born and after réttir*	Grazing area for ewes and lambs during the growing season (mid-June to mid-September)
Goat (*Capra hircus* L.)	Domestic, introduced	Grazing pastures during the growing season (May to Sept)	-
Greenland white fronted goose (*Anser albifrons flavirostris* S.)	Wild, native	Stopover during spring and autumn, before migrating to breeding sites in Greenland. Arrival peak passage between 20 and 25 April and early May	-
Greylag goose (*Anser anser* L.)	Wild, native	Staging and breeding area from spring to autumn. Arrival usually two weeks before pink footed geese and departure after the pink footed geese. A small part of the population recently became resident in Iceland (about 5%**)	-
Pink footed goose (*Anser brachyrynchus* B.)	Wild, native	Stopover during spring and autumn. Peak of arrival early May (8th of May). Part of the population continue the migration to Northeast Greenland breeding area in summer (perhaps 15–25%, Frederiksen et al. 2004)	Breeding area during summer (from mid-May to late August)
Brent goose (*Branta bernicla* L.)	Wild, native	Stopover during spring and autumn before migrating to breeding areas in northeast Greenland	-
Barnacle goose (*Branta leucopsis* B.)	Wild, native	Stopover during spring (i.e., end of April to late May) and autumn before migrating to breeding areas in East Greenland	-
Whooper swan (*Cygnus cygnus* L.)	Wild, native	Stopover during spring and autumn	Breeding area during summer
Rock ptarmigan (*Lagopus muta* M.)	Wild, native	–	Stay year-round
Reindeer (*Rangifer tarandus* L.)	Wild, introduced	–	Stay year-round

*The “réttir” corresponds to the collection of sheep from the rangelands after the summer

**Expert communication

Wild herbivores include feral reindeer (*Rangifer tarandus*), several species of geese, whooper swans (*Cygnus cygnus*) and rock ptarmigan (*Lagopus muta*). Reindeer were introduced in the seventeenth century and are confined to East Iceland’s rangelands, where they are managed as game (Þórisson 2018). Waterfowl, including geese and swans, migrate from their wintering grounds (i.e., England, Scotland, and Ireland) to improved pastures in Iceland during spring and autumn. Among them, the Greenland white fronted goose (*Anser albifrons flavirostris*), the brent goose (*Branta leucopsis*) and the barnacle goose (*Branta bernicla*) continue their migration to Greenland where they breed (Fox et al. 1983); others, mainly pink footed geese (*Anser brachyrhynchus*), move to unimproved highland areas during summer to breed (Fox et al. 1992). Ptarmigans inhabit rangelands year-round and move seasonally to higher elevation (Gardarsson 1971).

### Herbivore abundance data

A long-term herbivore abundance dataset for Iceland, spanning 11 vertebrate species (Table 1), was compiled from diverse published and unpublished sources with variable temporal and spatial coverage (Supplementary Material S1). Complete national population records for all species were available since 1986. Yearly livestock records since 1950, were retrieved from the national statistics database (Statistics Iceland 2022). Reindeer census data came from yearly aerial surveys in early July (Þórisson 2018). Waterfowl abundance data were derived from autumn colony censuses in UK wintering areas (Fox et al. 1998; Mitchell et al. 2010; Brides et al. 2021), as these estimates provide an accurate estimate of the abundance of waterfowl in Iceland during the summer months (Frederiksen et al. 2004). Most censuses were conducted yearly except for the barnacle geese and whooper swans, for which censuses were conducted every 5 years (Supplementary Material S1). One waterfowl species (the brent goose) was not included in the dataset due to lack of long-term consistent census data. Population estimates for the rock ptarmigan were based on biannual censuses (i.e., early May and early August, respectively, estimating summer and winter population) in East Iceland extrapolated to the entire country (Magnússon et al. 2004). Those estimates slightly overestimate the overall ptarmigan population (Sturludóttir 2015), but are nonetheless the longest and best available time series to date.

Herbivore densities were calculated by dividing abundance estimates by the total grazing area in Iceland (Stefánsson et al. 2020). The total grazing area is the sum of commonly and privately owned rangelands and improved lands. Full details on the database are provided in Supplementary Material S1.

### Metabolic biomass of wild and domestic herbivores

Metabolic biomass (MB) is an allometric function that represents an animal’s daily energy requirements. MB enables comparisons among species and allows estimating herbivore pressure in multi-species assemblages (Hatton et al. 2015). We calculated the species-specific metabolic biomass derived from Kleiber's (1932) equation based on the body mass (BW) and the metabolic rate (MR) of species i:

$${{\text{MB}}}_{i}={{{\text{BW}}}_{i}}^{{{\text{MR}}}_{i}}$$

As no data on the age or sex of individuals were available for most of the species, we used the average mass of an adult obtained from the literature (Supplementary Material S2). For the metabolic rate, we used species-averaged estimates for birds and mammals (i.e., respectively 0.71 and 0.64; Hudson et al. 2013).

Population level estimates of metabolic biomass (PMB_i_, kg·year^−1^) were calculated multiplying the species-specific metabolic biomass, by the abundance of the species in a given year and scaled by the amount of time spent grazing per year (*G*_i_; *G*_i_ ranges from 0 to 1, 1 being for species grazing all year round and less for species that use the grazing areas seasonally; Supplementary Material S2).

PMB values were summed across species for the entire herbivore community (PMB_total_), and for wild and (PMB_wild_) and domestic (PMB_livestock_) species separately and standardized by the sum of grazing areas in kilometre squares.

### Forage intake of wild and domestic herbivores in improved and unimproved land

Dry matter forage intake (DMI; DM kg year^−1^) was used to estimate plant consumption by wild and domestic herbivores in improved lands and rangelands. Livestock DMI values were calculated following Holecheck (1998), using a value of 2% of the animals’ body mass (kg) for ruminants and 3% for horses. DMI values of wild birds (waterfowl and ptarmigan) were extracted from literature. Data were either Iceland-specific, from comparable arctic or subarctic environments, or from closely related species (Supplementary Material S2). DMI values were multiplied by the abundance of each species and the number of days spent grazing in either improved lands or rangelands separately. Values were summed across the herbivore community (DMI_total_), livestock (DMI_livestock_) and wild species (DMI_wild_) and were standardized by the area of each type of land.

DMI was converted to carbon-based units (kg C year^−1^ km^−2^), assuming that plants contain 45% of carbon (C) on average (Ma et al. 2018). We compared those values with yearly net primary production (NPP, in kg C year^−1^ km^−2^) obtained from MODIS MOD 17 satellite derived products (Running and Zhao 2021) available from 2000 to 2021. We computed the average NPP for each year separately for improved lands and rangelands, using the map of grazing areas in Iceland (Stefánsson et al. 2020), and estimated the proportion of NPP consumed by wild and domestic herbivores in each type of land.

### Accounting for parameter uncertainties

The calculations presented above are based on best available estimates of the parameters in the equations. Yet, most sources did not report a measure of variability, although these parameters are known to vary. To consider this variability, we simulated 100 replicates where each parameter varied stochastically by being perturbed by 10% from the baseline values, assuming a normal distribution (Supplementary Material S3).

### Data analysis

Changes over time in herbivore community composition were analysed using Nonparametric Multidimensional Scaling (NMDS). We computed a Bray–Curtis dissimilarity matrix based on the density of each herbivore species in each year. Species composition was plotted using two axes to visualize temporal changes between consecutive years (Matthews et al. 2013).

Generalized Additive Models (GAM, Hastie and Tibshirani 1990) were employed to analyse temporal trends of species-specific densities, metabolic biomass, and forage intake. GAMs allow for the detection of nonlinear trends in time series data while ignoring fine-scale bias (Fewster et al. 2000). Year was modelled as a cubic spline to account for interannual variation. Herbivore densities were modelled assuming a gamma distribution which is appropriate for positive continuous variables; other response variables were modelled as Gaussian (Supplementary Material S4). Net changes in herbivore densities, metabolic biomass and forage intake were assessed after predicting each variable during the first (1986) and the last year (2020) of records. While this analysis blurs the nonlinear dynamics, it provides an overview of net changes in population sizes. Differences in forage intake within improved and unimproved lands were assessed with a *t* test.

All statistical analyses were carried out in R version 4.2.3 (R Core Team 2023). The packages *mass* (Ripley et al. 2013) and *vegan* (Oksanen et al. 2013) were used to run the NMDS, and *mgcv* (Wood and Wood 2015) for GAM. Unless stated otherwise, mean values and standard errors are presented.

## Results

### Changes in herbivore community composition

**Fig. 1:**
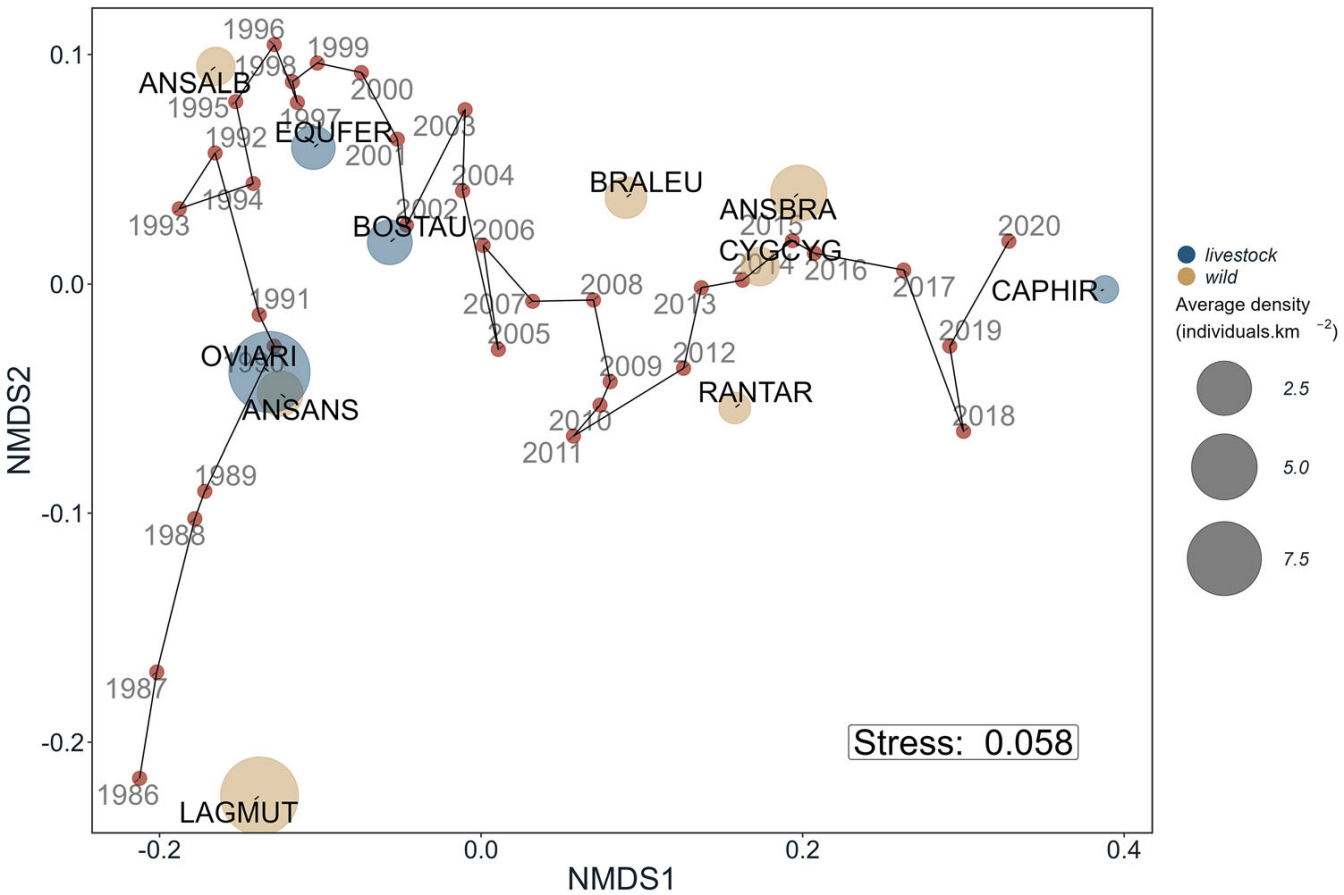
NDMS visualizing changes in herbivore community composition in Iceland from 1986 to 2020. The solid line represents the trajectory of the overall herbivore community between years. Blue points are livestock species and yellow are wild species. Size of the points represents the average density (individuals km^−2^) of each species. LAGMUT = rock ptarmigan, OVARI = sheep, ANSANS = Greylag goose, ANSALB = White fronted goose, EQUFER = horse, BOSTAU = cattle, BRALEU = Barnacle goose, CYGCYG = whooper swan, RANTAR = reindeer, ANSBRA = pink footed goose, CAPHIR = goats

From 1986 to early 2000, herbivore community composition was constrained to the left of the axis 1 of the NMDS plot (Fig. 1), with a clear directional trend from the bottom to the top of axis 2. After 1999, the trajectory moved downward on axis 2 and towards the right, parallel to axis 1. Species broadly clustered in two main groups across the biplot, with most livestock species (sheep, horses, and cattle) located at the left of the plot, and most wild species (barnacle goose, whooper swan, pink footed goose and reindeer) located towards the central part of the plot.

### Changes in the densities of herbivores

**Fig. 2:**
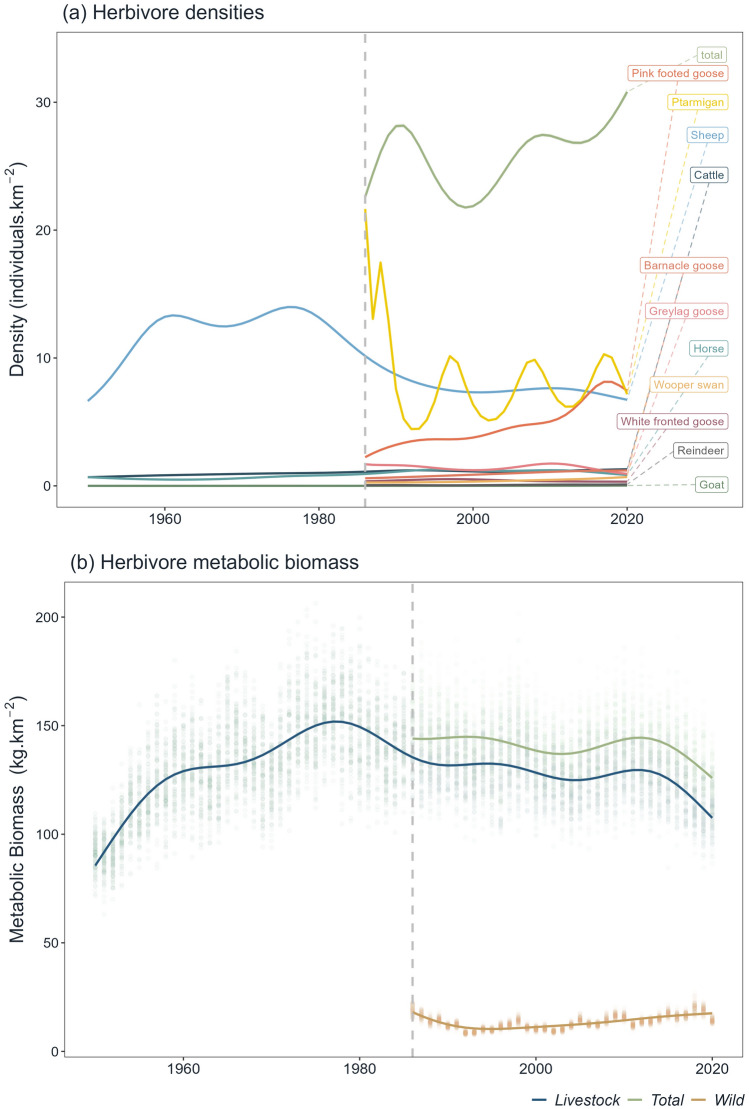
Changes in the densities of the main vertebrate herbivore species (**a**) and in metabolic biomass of domestic and wild herbivores (**b**) in Iceland between 1950 and 2020. Green curves indicate the pooled density (**a**) and pooled metabolic biomass (**b**) of all herbivore species. Vertical grey dashed lines in 1986 indicate the starting point when data for all species becomes available. Coloured curves result from GAM. Light coloured surfaces represent the standard error of the GAM estimates and the points represent estimates of metabolic biomass, resulting from the uncertainty analysis

Between 1986 and 2020, when records were available for all species, total herbivore density significantly decreased by 29.30% (Supplementary Material S4, Fig. 2a). The estimated density of herbivores declined from 37.60 ± 0.06 individuals km^−2^ in 1986 to 26.60 ± 0.06 individuals km^−2^ in 2020, reaching a minimum in 1993 with 20.60 ± 0.04 individuals km^−2^.

Population densities of the 11 species analysed displayed significant fluctuations between 1986 and 2020. Most herbivore populations increased, except for the sheep, white fronted goose, greylag goose and ptarmigan (Supplementary Material S5 and S6). Among livestock, sheep remained the most abundant herbivore in Iceland throughout the time series. By 2020, sheep constituted 25% of the total herbivore density contrasting with 4.89%, 3.17%, 0.09% for cattle, horses and goats, respectively. Sheep densities peaked at 13.90 ± 0.07 individuals km^−2^ in 1978, prior to the implementation of the livestock quota in the 1980s. Ptarmigan exhibited densities comparable to sheep, but fluctuated greatly over time, with a peak density of 21.90 ± 0.28 individuals km^−2^ in 1986. In turn, the density of pink footed goose increased from 2.21 ± 0.03 individuals km^−2^ in 1950 to 7.54 ± 0.08 individuals km^−2^ in 2020, consolidating its position as one of the most abundant herbivores, representing 29.10% of the total herbivore density in 2020.

### Changes in metabolic biomass of wild and domestic herbivores

Total herbivore metabolic biomass (PMB_total_) decreased by 19.40% (Fig. 2b, Supplementary Material S6), declining from a peak of 154.30 ± 0.31 kg km^−2^ in 1986 to 124.40 ± 0.31 kg km^−2^ in 2020. This decrease was primarily driven by changes in livestock metabolic biomass. Specifically, PMB_livestock_ decreased by 20.40%, from 135.30 ± 0.18 kg km^−2^ in 1986 to 107.60 ± 0.34 kg km^−2^ in 2020 with an average of 130 kg km^−2^ over the entire period. In contrast, PMB_wild_ varied substantially during this time, but remained considerably lower than PMB_livestock_ throughout the period, with an average of 13.50 ± 1.06 kg km^−2^. Current values (17.50 ± 0.83 kg km^−2^) are commensurate to estimates from 1986 (18.10 ± 0.91 kg km^−2^).

Before 1986, data were only available for PMB_livestock_. In 1950, values of PMB_livestock_ were comparable to those in 2020 (85.50 ± 0.76 kg km^−2^ and 107 ± 0.76 kg km^−2^, respectively). However, PMB_livestock_ underwent significant changes over this interval, peaking in 1978 at the historical maximum of 151.70 ± 0.41 kg km^−2^.

### Forage intake of wild and domestic species

**Fig. 3:**
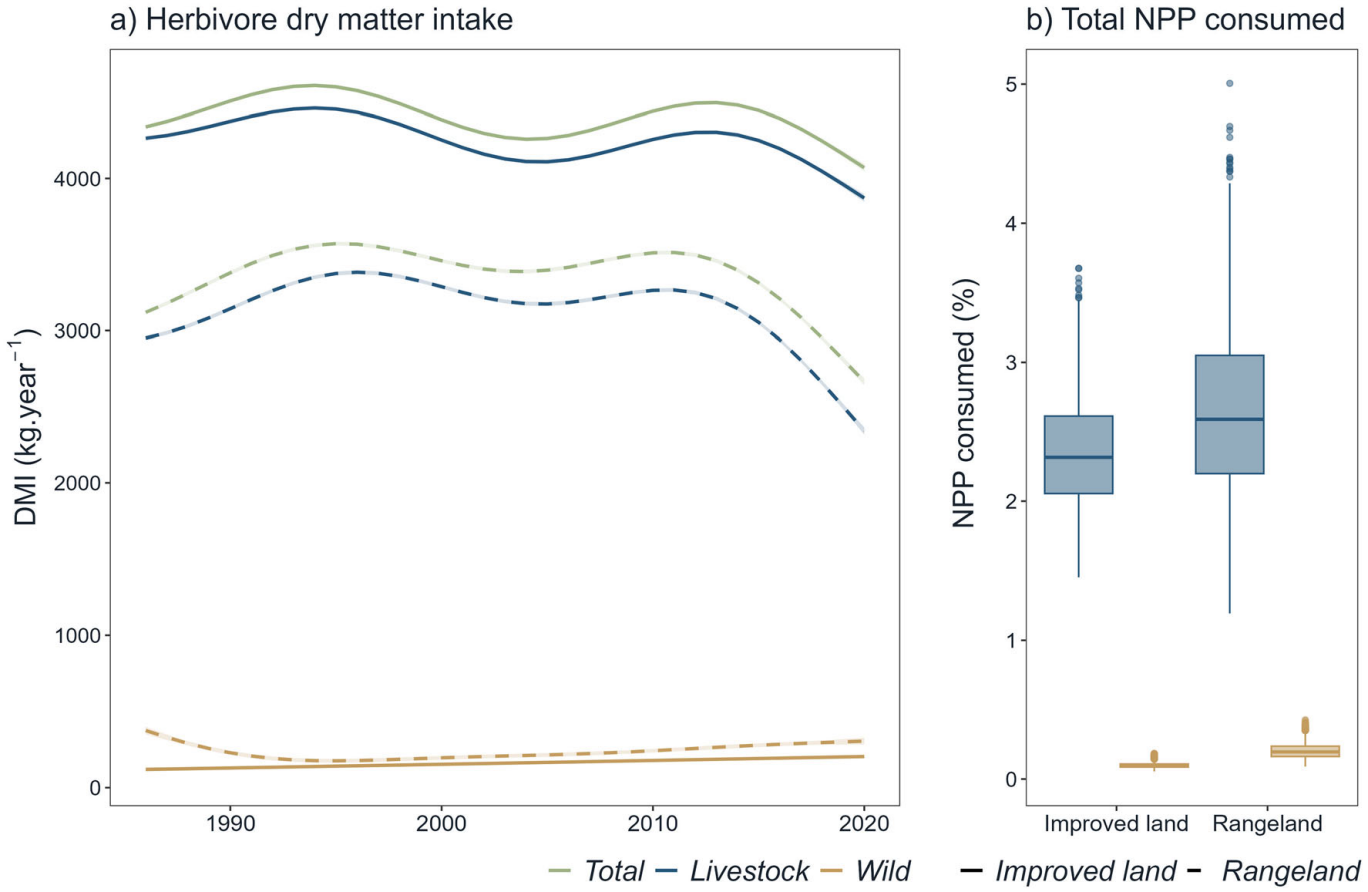
Wild and domestic herbivore dry matter intake between 1986 and 2020 (**a**), and proportion of plant biomass consumed by wild and domestic herbivores between 2000 and 2020 on Icelandic grazing areas (**b**). Coloured smooth curves in (**a**) result from GAM

Total forage intake by herbivores (DMI_total_) between 1986 and 2020 was significantly higher in improved lands than in rangelands (*t* test; *t* = 87.4, df = 684.55, *p* value < 0.001). Overall, forage intake significantly changed in each land type and species group during the study period (Supplementary Material S4). Total forage intake decreased in both rangelands and improved land (Fig. 3a) but the magnitude of the decline was greater in rangelands (14.60% vs. 6.16%).

When considering each group of herbivores separately, forage intake by livestock decreased between 1986 and 2020 in both land types, and this decline was stronger in rangelands (20.60% vs. 9.22%: Fig. 3a). The estimated intake by wild species also decreased in rangelands by 18.60% (from 373 ± 24.4 kg C year^−1^ km^−2^ in 1986 to 304 ± 22.4 kg C year^−1^ km^−2^ in 2020), but increased in improved lands by 72.50% (from 118 ± 13.2 kg C year^−1^ km^−2^ in 1986 to 202 ± 13.2 kg C year^−1^ km^−2^ in 2020). Still, dry matter intake by wild species remained overall consistently low compared to DMI_livestock_, as it represented on average 3.62% of the total herbivore consumption in improved lands and 7.13% in unimproved lands.

Total net primary production (NPP) was on average higher in improved land compared to rangeland (mean values recorded from 2000 to 2020 were, respectively, 179 421 ± 7771 kg C km^−2^ year^−1^and 118 905 ± 6136 C km^−2^ year^−1^). Between 2000 and 2020, the NPP increased in improved lands and rangelands by, respectively, 10.50% and 8.00%, but NPP was more variable in improved lands (sd = 20 560 vs. sd = 16 235) (Supplementary Material S7). When put in perspective with the estimates of average forage intake by herbivores, total wild herbivore consumption only accounted for 0.10 ± 0.01% and 0.21 ± 0.02% in improved grasslands and rangelands, respectively. In comparison, livestock consumed on average 2.35 ± 0.13% to 2.65 ± 0.20% of the total primary productivity (Fig. 3b), in improved and unimproved areas.

## Discussion

This study pioneers an assessment of long-term shifts in Iceland’s herbivore community, juxtaposing managed improved grasslands and rangelands. We observed an initial transition from a livestock-dominated to a wild-dominated community since 1986, in tune with ongoing farmer’s concerns. Yet, by 2020, wild species still contributed only 13.9% of total herbivore biomass, with livestock forage intake far surpassing that of wild herbivores. Interestingly, our analyses indicate a decline in herbivore forage intake since 1986, particularly in rangelands. This may be due to a higher dependency on cultivated and imported fodder, which has been the tendency in the past decades (Helgadóttir and Hopkins 2013). Conversely, wild herbivore consumption in improved lands increased, yet their overall intake remained modest.

Results from NMDS suggested a shift from livestock to wildlife dominance by the mid-1990s and a diversification of livestock. This shift aligns with global trends in waterfowl population growth, notably the pink footed goose, alongside with local livestock management shifts, like the implementation of a livestock quota in the 1980s and renewed efforts for the conservation of goat breeds (Dýrmundsson 2005). Similar transitions from livestock to wild dominance have been documented in other Nordic countries, like Norway, where a recovery of large wild ungulates and a decline in domestic species has been reported (Austrheim et al. 2011). These transitions have been interpreted as trophic rewilding (Speed et al. 2019). While the observed changes in herbivore densities indicate that trophic rewilding might have also happened in Iceland, those results do not hold in terms of metabolic biomass, where the metabolic biomass of wild herbivores remained much lower than that of livestock. A potential explanation could be the limited number of large ungulates in Iceland which could increase the biomass of wild herbivore species in response to the decrease in livestock biomass (Austrheim et al. 2011).

The total average metabolic biomass of herbivores in Iceland (141 ± 2.03 kg km^−2^ in 2020) ranks among the lowest values recorded in Europe, including both livestock and wild species (190–16 000 kg km^−2^) (Fløjgaard et al. 2021) but is commensurate to what is observed in other high latitude ecosystems (108 kg km^−2^ in 2015, Speed et al. 2019). Herbivore metabolic biomass in Iceland has declined since 1986; largely driven by domestic herbivores. Assuming that this pattern was maintained before 1986, it is likely that Iceland had reached a maximum value of metabolic biomass in the late 70 s, when the densities of livestock species peaked. Given the short evolutionary history of grazing in Iceland, where mammalian herbivores were introduced in historical times, this might have significant ecological implications as land recovery is usually limited in areas characterized by a short grazing history (Price et al. 2022).

Livestock consumed more plant biomass than wild species, but their forage intake remained far below than in other Northern European rangelands (1.43–5.36% vs. 18.6–24.3%, Wolf et al. 2021). Wild species consumed on average less than 0.3% of the net primary production, while livestock consumed approximately 2% in both land types, which is in stark contrast to global averages (11% NPP for wild mammals; Pedersen et al. 2020). Total herbivore biomass consumption decreased, likely driven by livestock reduction, which was modest in improved lands, therefore relaxing the historical pressure exerted in those lands.

### Consequences for management

This study sheds light on shifting herbivore pressure across two distinct land types: improved grasslands, extensively modified by human activities, and rangelands, managed as more natural ecosystems. As such, the management implications of changing herbivore dynamics in these areas differ significantly.

Improved grasslands, where most livestock are grazed, have expanded over the last century due to agricultural advancements and expansion of government subsidies (Wald 2010; Helgadóttir and Hopkins 2013). Enhancements like nitrogen-based fertilizers have boosted grass nutritional quality and crop yields, benefiting both livestock production and waterfowl populations (Jefferies et al. 2003; Fox and Abraham 2017). However, increasing waterfowl densities on improved lands can lead to conflicts between wild and livestock species. Such tensions can arise due to perceived competition and the greater investments that farmers make on improved pastures compared to rangelands, and have been reported from grazing areas worldwide (Baldi et al. 2001; Mason et al. 2018). While culling and financial compensation are proposed solutions (Eythórsson et al. 2017), interactions between wild and domestic species, particularly via vegetation, can complicate management strategies. As waterfowl often arrive before livestock are released in the pastures and they form large flocks during migration, they might have concentrated impacts and affect the amount of vegetation and plant species available later (Bjerke et al. 2021). However, some studies have shown that geese do not interfere with livestock feeding and may not reduce the yield of fertilized pastures, but can instead increase nutrient availability for grasses (Gorosábel et al. 2019). Investigating potential positive interactions between these species, especially considering the lower impact of geese compared to livestock, warrants further research.

In contrast, rangelands, managed with less intensity, endure harsh climates, and exhibit lower productivity. These areas are more sensitive to disturbances compared to improved grasslands and are often deemed to be in poor condition (Arnalds et al. 2023). Grazing practices (e.g., length of grazing period and stocking density) vary among grazing commons (Arnalds and Barkarson 2003). While concerns of conflict akin to those in improved lands exist, our findings suggest that despite the spatial overlap between wild and domestic species (Boulanger-Lapointe et al. 2022) wild species have a moderate consumption of plant biomass in rangelands. Similar observations in Norwegian rangelands point to limited competition in low density, unfenced settings (Speed et al. 2019). Facilitation, rather that competition, is more plausible in low-productive ecosystems (Barrio et al. 2013). In turn, reduced herbivore pressure in rangelands presents an opportunity for their restoration coupled with strategic management changes (Mulloy et al. 2019).

### Knowledge gaps identified

While this study encompasses an extensive temporal scale, spanning over 70 years for several herbivore species, and represents the most comprehensive long-term dataset available for terrestrial herbivores in Iceland, certain limitations should be acknowledged. Our analyses addressed the inherent uncertainties associated with the compilation of data from diverse sources. Despite these uncertainties, it is noteworthy that our results demonstrated consistent patterns in herbivore community changes, metabolic biomass, and herbivore dry matter forage intake. Our assumptions reveal important data gaps for the study of herbivore community dynamics in high-latitude managed grazing systems.

Information related to herbivore land use, including finer scale data on spatial and temporal use remain very scarce and poorly monitored for many waterfowl (Arzel et al. 2006). Similarly, systematic efforts are needed to collect and synthesize data from local and regional sources for livestock species. Further research and monitoring will allow exploring approaches based on finer spatial and temporal resolution that could reveal areas or periods more prone to wildlife conflicts, where wild and domestic species coexist. Such analyses will provide information on the potential interactions between species and help disentangling management conflicts by locally adapting management practices.

## Conclusions

Wildlife and livestock conflicts occur in many places around the world, including Iceland. Yet, studies exploring the relative impact of wild species compared to livestock remain scarce. The long population records available in Iceland allowed us to evaluate the potential vegetation impact of domestic and wild herbivores at a national scale. Consistent long-term spatially and temporally explicit data might be the key to further solve these issues. Stakeholders play a valuable role in providing such information, and this paper stands as a call to develop collaborations.

### Supplementary Information

Below is the link to the electronic supplementary material.

Supplementary file1 (PDF 1485 KB)
