# Supplementary material for: Long-term changes in herbivore community and vegetation impact of wild and domestic herbivores across Iceland
Source: Ambio. 2024 Feb 25;53(8):1124–35. doi: 10.1007/s13280-024-01998-6 (PMC11182994; doi:10.1007/s13280-024-01998-6)
Supplement: Supplementary file 1 — Supplementary file1 (PDF 1485 KB) [file 13280_2024_1998_MOESM1_ESM.pdf]

Title: Long-term changes in herbivore community and vegetation impact of wild and domestic herbivores across Iceland

Mathilde Defourneaux[1]*, Isabel C. Barrio[1], Noémie Boulanger-Lapointe[2], James D.M. Speed[3]

[1]Faculty of Environmental and Forest Sciences, Agricultural University of Iceland, Árleyni 22, Keldnaholt, 112 Reykjavík, Iceland

[2]Department of Geography, University of Victoria, Victoria, BC, Canada

[3]Department of Natural History, NTNU University Museum, Norwegian University of Science and Technology, 7491 Trondheim, Norway

*corresponding author: mathilde@lbhi.is

Word count: 6 217 words

Acknowledgements:

We would like to thank Ólafur K. Nielsen and Erla Sturludóttir for ptarmigan population data, Skarphéðinn Þórisson for reindeer census data; Carl Mitchell, Antony D. Fox, and Kane Bride for waterfowl census data; and Landgræðslan for sharing grazing area data and Emmanuel P. Pagneux for his help with MODIS data. Funding for this project was provided by the Icelandic Research Fund (grant nr. 217754; TUNDRAsalad). This paper is dedicated to the memory of Skarphéðinn Þórisson who tragically passed away in July 2023.

Author contributions

Conceptualization: M. Defourneaux, I. C. Barrio, J. D.M Speed, N. Boulanger-Lapointe; Methodology: M. Defourneaux, I. C. Barrio, J. D.M Speed, N. Boulanger-Lapointe; Formal analysis and investigation: M. Defourneaux; Writing - original draft preparation: M. Defourneaux; Writing - review and editing: I. C. Barrio, J. D.M. Speed, N. Boulanger-Lapointe; Funding acquisition: I. C. Barrio; Supervision: I. C Barrio, J. D.M. Speed, N. Boulanger Lapointe

Conflict of interest

The authors have no relevant financial or non-financial interest to disclose.

Ambio

Supplementary Information

This supplementary information has not been peer reviewed.

Title:

Long-term changes in herbivore community and vegetation impact of wild and domestic herbivores across Iceland

Supplementary material

Supplementary material S1. Population data for herbivore species in Iceland

Supplementary material S2. Estimates of body mass, forage intake and grazing time for each herbivore species obtained from the literature

Supplementary material S3. Evaluation of the uncertainty of the metabolic biomass and herbivore forage intake estimates

Supplementary material S4. Results from the Generalized Additive Models

Supplementary material S5. Species-specific herbivore density trends

Supplementary material S6. Estimated net changes between 1986 and 2020

Supplementary material S7. Averaged yearly net primary productivity within improved lands and rangelands in Iceland from 2000 to 2020

Supplementary material S1. Population data for herbivore species in Iceland

Domestic species

Livestock numbers during winter (i.e., sheep, horses, cattle, and goats) were retrieved from national statistic records[1]. Demographic groups encompassed the total number of individuals for each herbivore species, including scarce details regarding young (<1 year old), adult males (>1 year old for sheep, 4 years old for horses), female and yearling (grouped), and non-reproductive individuals.

Reindeer

Reindeer census data were derived from aerial surveys conducted yearly in early July every by the East Iceland Nature Research Centre (Náttúrustofa Austurlands)[2]. Census categories include young (1-2 years old) and adult males (>3 years), females and yearlings (indistinguishable), and calves (2 months).

Waterfowl

Waterfowl abundance data were obtained from the World Waterfowl Trust (**Table S1**). Annual colony censuses were conducted in October-November in the wintering areas in the UK (i.e., Scotland, Ireland) for Greenland white fronted geese, greylag and pink footed geese[3–5]. Censuses for barnacle geese and whooper swan occurred every 5 years and were done by aerial surveys. These counts encompassed returning adults and some non-migratory individuals residing year-round in the UK. Still, these colony censuses represent an unbiased estimate of the abundance of waterfowl present in Iceland during the summer months[6]. One waterfowl species (i.e., the brent goose, *Branta bernicla*) was not included in this study due to the lack of long-term consistent census data. However, the population was estimated to be relatively stable from the late 60s to 2000 (from 8000-13 000 individuals 18 000- 24 000)[7], and to slightly

increase until 2014 (32 000 individuals, or a density of 0.5 individuals.km^{-2})[8], representing 5.2% of the overall waterfowl population in Iceland.

Rock ptarmigan

Population estimates for the rock ptarmigan were provided by the Icelandic Institute of Natural History. They were extrapolated from annual census conducted in East Iceland to represent the entire country population (Magnússon et al., 2004). Biannual censuses in early May and early August from 1981 to 2021 approximated summer and winter populations. For our study, we used a yearly average based on both estimates standardized by the number of months in summer (3) and in winter (9). Those estimates slightly overestimate the overall ptarmigan population [10], but are nonetheless the longest and best available time series to date.

Table S1. List of herbivores in Iceland and dataset descriptors of their abundance. Time period and frequency respectively correspond to the first and last year of population census records and the frequency of population census.

Species	Time period	Frequency	References
Greenland white fronted goose (*Anser albifrons flavirostris)*	1983-2020	Yearly	[3]
Greylag goose (*Anser anser)*	1953-2020	Yearly	[4]
Pink footed goose (*Anser brachyrynchus)*	1950-2020	Yearly	[4]
Brent goose (*Branta bernicla*)	NA	NA	NA
Barnacle goose (*Branta leucopsis)*	1959-2020	5 years	[4]
Whooper swan (*Cygnus cygnus)*	1986-2020	5 years	[5]
Rock ptarmigan (*Lagopus muta)*	198-2020	Yearly	[9]
Reindeer (*Rangifer tarandus)*	1964-2020	Yearly	[2]
Cattle (*Bos taurus)*	1950-2020	Yearly	[1]
Icelandic Horse (*Equus ferus caballus)*	1950-2020	Yearly	[1]
Icelandic sheep (*Ovis aries)*	1950-2020	Yearly	[1]
Goat (*Capra hircus)*	1950-2020	Yearly	[1]

References:

1. Statistics Iceland. Agricultural products. (2022) doi:https://sogulegar.hagstofa.is/landbunadur/.

2. Þórisson, S. G. Population dynamics and demography of reindeer (*Rangifer tarandus* L.) on the East Iceland highland plateau : 1940–2015 A comparative study of two herds. (Agricultural University of Iceland, 2018).

3. Fox, A. D., Gitay, H., Owen, M., Salmon, D. G. & Ogilvie, M. A. Population dynamics of Icelandic-nesting geese, 1960-1987. *Ornis Scandinavica* **20**, 289 (1989).

4. Mitchell, C. *et al.* Trends in goose numbers wintering in Britain & Ireland, 1995 to 2008. *Ornis Svec* **20**, (2010).

5. Brides, K. *et al.* The Icelandic whooper swan *Cygnus cygnus* population: current status and long-term (1986–2020) trends in its numbers and distribution. *Wildfowl* 29–57 (2021).

6. Frederiksen, M. *et al.* The dynamics of hunted Icelandic goose populations: a reassessment of the evidence. *J Appl Ecology* **41**, 315–334 (2004).

7. Alisauskas, R. T. Goose populations of the Western Palearctic: a review of status and distribution. *The Auk* **117**, 271–272 (2000).

8. Fox, A. D. & Leafloor, J. O. *A global audit of the status and trends of Arctic and Northern hemisphere goose populations (Component 2: Population accounts)*. 31 (2018).

9. Magnússon, K. G., Brynjarsdótti, J. & Nielsen, O. K. *Population cycles in rock ptarmigan Lagopus muta: modelling and parameter estimation.* 36 (2004).

10. Sturludóttir, E. Statistical analysis of trends in data from ecological monitoring. (University of Iceland, 2015).

Supplementary material S2. Estimates of body mass, forage intake and grazing time for each herbivore species obtained from the literature

Literature searches for relevant data on body mass, forage intake and grazing time were carried out on scientific search engines (Web of Science, Google Scholar, Scopus) on December 2022. We searched for common name (full e.g., pink footed goose and short e.g., goose, swan) or Latin names of the species, in addition to the expressions "*Iceland*" or "**arctic*" or "*North**, and "*forage intake*" or "*DMI*" or "*dry matter intake*" or "*offtake*" or "*body weight*". For Icelandic breed-specific information we added "*Iceland*" or "*Icelandic breed*".

For grazing time on improved pasture and rangelands, qualitative information was prevalent, especially for livestock species. Information on timing of year-to-year livestock release to pastures and waterfowl arrival were truly lacking in the literature, as well as regional variations.

Due to the absence of local forage intake data of free-ranging animals and the specificities of Icelandic mammal breeds, values of daily dry matter intake (DMI) for mammals (livestock and feral reindeer) were estimated following Holecheck (1998). This involved assigning a value of 2% of body mass for ruminants and 3% for horses. DMI values of wild birds (waterfowl and ptarmigan) were extracted from the literature, either directly from the species in Iceland, from the same species in a comparable arctic or subarctic environment, or from a closely related species when no species-specific data were available. For example, DMI values for graylag and Greenland white-fronted geese were based on data from pink footed goose, a species that has a similar diet and body weight.

Table S2. Adult averaged species-specific values for body weight (BW), dry matter intake (DMI), and grazing time (total Gtot, improved lands Gimp and rangelands Grang). These variables correspond to the average adult body mass, the amount of dry vegetation ingested within a day (in kg.day^{-1}), the proportion of days spent grazing within a year and the number of days spent grazing in improved grasslands and rangelands.

Species name	BW (kg)	DMI (kg.day^{-1})	Gtot (day and proportion)	Gimp/Grang (days)	References
Greenland white fronted goose (*Anser albifrons flavirostris*)	2.64	0.20	71 (0.19)	71 / 0	[2–4]
Greylag goose (*Anser anser)*	3.30	0.20	178 (0.49)	178 / 0	[4–8]
Pink footed goose (*Anser brachyrynchus)*	2.51	0.20	160 (0.44)	68 / 92	[4–7,9,10]
Barnacle goose (*Branta leucopsis)*	1.68	0.15	75 (0.21)	75 / 0	[4,11–15]
Whooper swan (*Cygnus cygnus)*	9.35	0.24	183 (0.50)	91 / 92	[4,16,17]
Rock ptarmigan (*Lagopus muta)*	0.50	0.06	365 (1.00)	0 / 365	[18,19]
Reindeer (*Rangifer tarandus)*	80	1.60	365 (1.00)	0 / 365	[20]
Cattle (*Bos taurus)*	470	9.40	123 (0.34)	123 / 0	[21–23]
Icelandic Horse (*Equus ferus caballus)*	379	11.4	365 (1.00)	109 / 256	[24–26]
Icelandic sheep (*Ovis aries)*	65	1.30	184 (0.5)	92 / 92	[27]
Goat (*Capra hircus)*	56.25	1.12	123 (0.34)	123 / 0	[28]

Refences:

1. Holecheck, J. W. An approach for setting the stocking rate. *Rangelands* **10**, (1998).

2. Fox, A. D., Madsen, J. & Stroud, D. A. A review of the summer ecology of the Greenland whitefronted goose *Anser albifrons flavirostris*. *Dansk Ornitologisk Forenings Tidsskrift* 43–55 (1983).

3. Budeau, D. A., Ratti, J. T. & Ely, C. R. Energy dynamics, foraging ecology, and behavior of prenesting greater white-fronted geese. *The Journal of Wildlife Management* **55**, 556 (1991).

4. Wilman, H. *et al.* EltonTraits 1.0: Species-level foraging attributes of the world's birds and mammals: *Ecological Archives* E095-178. *Ecology* **95**, 2027–2027 (2014).

5. Fox, A. D., Gitay, H., Owen, M., Salmon, D. G. & Ogilvie, M. A. Population dynamics of Icelandic-nesting geese, 1960-1987. *Ornis Scandinavica* **20**, 289 (1989).

6. Frederiksen, M. *et al.* The dynamics of hunted Icelandic goose populations: a reassessment of the evidence. *J Appl Ecology* **41**, 315–334 (2004).

7. Fox, A. D., Gitay, H., Boyd, H. & Tomlinson, C. Snow-patch foraging by pink-footed geese *Anser brachyrhynchus* in south Iceland. *Ecography* **14**, 81–84 (1991).

8. Fox, A. D., Boyd, H. & Warren, S. M. Spatial and temporal feeding segregation of two Icelandic goose species during the spring pre-nesting period. *Ecography* **15**, 289–295 (1992).

9. Madsen, J. Relations between change in spring habitat selection and daily energetics of pink-footed geese *Anser brachyrhynchus*. *Ornis Scandinavica* **16**, 222 (1985).

10. Therkildsen, O. R. & Madsen, J. Assessment of food intake rates in pink-footed geese *Anser brachyrhynchus* based on examination of oesophagus contents. *Wildlife Biology* **6**, 167–172 (2000).

11. Ebbinge, B., Canter, K. & Drent, R. Foraging routines and estimated daily food intake in barnacle geese wintering in the northern Netherlands. *Wildfowl* **26**, 5–19 (1975).

12. Owen, M., Wells, R. L. & Black, J. M. Energy budgets of wintering barnacle geese: the effects of declining food resources. *Ornis Scandinavica* **23**, 451 (1992).

13. Tinkler, E., Montgomery, W. I. & Elwood, R. W. Foraging ecology, fluctuating food availability and energetics of wintering brent geese. *Journal of Zoology* **278**, 313–323 (2009).

14. Percival, S. M. & Percival, T. Feeding ecology of barnacle geese on their spring staging grounds in northern Iceland. *Ecography* **20**, 461–465 (1997).

15. Doyle, S. *et al.* Temperature and precipitation at migratory grounds influence demographic trends of an Arctic-breeding bird. *Glob. Change Biol.* **26**, 5447–5458 (2020).

16. Brazil, M. A. The behavioural ecology of whooper swan. (University of Stirling, 1981).

17. Bulstrode, C. J. K., Corbett, E. S. & Putnam, R. J. Breeding of whooper swans in Iceland. *Bird Study* **20**, 37–40 (1973).

18. Nielsen, O. K., Weiss, A. & Guðmundsson, G. A. *Rock ptarmigan (Lagopus muta) health studies in Northeast Iceland 2013: morphology and body reserves*. (2014).

19. Gardarsson, A. Food ecology and spacing behavior of rock ptarmigan (*Lagopus mutus*) in Iceland. (University of California, 1971).

20. Þórisson, S. G. Population dynamics and demography of reindeer (*Rangifer tarandus* L.) on the East Iceland highland plateau : 1940–2015 A comparative study of two herds. (Agricultural University of Iceland, 2018).

21. Sigurdsson, A. & Jonmundsson, J. V. Genetic potential of Icelandic dairy cattle. *ICEL. AGRIC. SCI.* 55–64 (2011).

22. Fridriksson, S. Grass and grass utilization in Iceland. *Ecology* **53**, 785–796 (1972).

23. Hersteinsson, P. & Hersteinsson, P. Mammals of the Thingvallavatn Area. *Oikos* **64**, 396 (1992).

24. Hoffmann, G. *et al.* Estimation of the body weight of Icelandic horses. *Journal of Equine Veterinary Science* **33**, 893–895 (2013).

25. Magnússon, S. & Magnusson, B. Studies in the grazing of a drained lowland fen in Iceland. II. Plant preferences of horses during summer. *Icel Agr Sci* 109–124 (1990).

26. Gudmundsson, O. & Dyrmundsson, O. R. Horse grazing under cold and wet conditions: a review. *Livestock Production Science* **40**, 57–63 (1994).

27. Ross, L. C. *et al.* Sheep grazing in the North Atlantic region: A long-term perspective on environmental sustainability. *Ambio* **45**, 551–566 (2016).

28. Dýrmundsson, Ó. R. The Iceland goat: past and present. *Anim. Genet. Resour. Inf.* **36**, 53–59 (2005).

Supplementary material S3. Evaluation of the uncertainty of the metabolic biomass and herbivore forage intake estimates

The analyses presented in this study are based on best available estimates for the parameters in the equations. Although these parameters (e.g., the average body mass of a species of herbivore or the time that animals graze on each type of land) are known to vary, most sources did not report a measure of variability. Most records for population size, body mass and grazing time reported a single value with no associated estimate of variability. To account for parameter uncertainty in our analyses, we simultaneously estimate 100 replicates of each parameter (i.e., in the case of metabolic biomass estimates, parameters were: body weight (BW_i), number of individuals (N_i) and total grazing time (G_i); for forage intake estimates, parameters were: dry matter intake (DMI_i), and grazing time in improved lands ($Gimp_i$) and rangelands ($Grang_i$)) by perturbing the baseline levels reported in **Supplementary material S1** by 10%, assuming that variables were normally distributed. After computing the PMB and DMI at the species level, we estimated the metabolic biomass and the forage intake for the total, domestic and wild herbivore species separately by conducting 100 iterations, with resampling, to generate estimates for each year in the period from 1950 to 2020. We calculated the standard error for these estimates within each herbivore group and year. Additionally, we estimated the yearly lower and upper limits of the estimates by summing maximum and minimum values of the PMB and DMI across each herbivore group (**Figure S3**). Uncertainty estimates were run on R version 4.2.3 (R Core Team 2023).

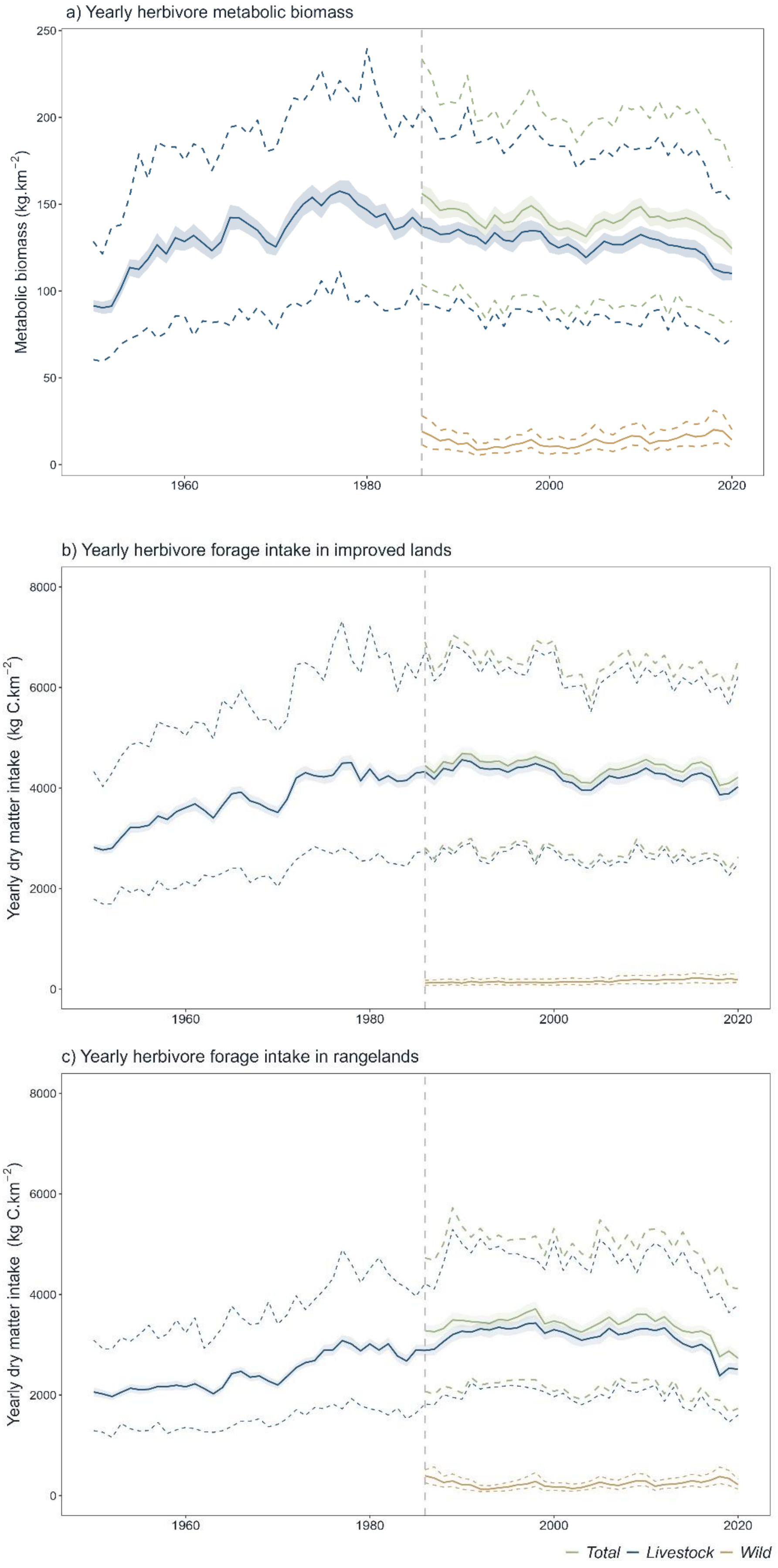
a) Yearly herbivore metabolic biomass
Metabolic biomass (kg.km^{-2})
250
200
150
100
50
0
1960
1980
2000
2020
b) Yearly herbivore forage intake in improved lands
Yearly dry matter intake (kg C.km^{-2})
8000
6000
4000
2000
0
1960
1980
2000
2020
c) Yearly herbivore forage intake in rangelands
Yearly dry matter intake (kg C.km^{-2})
8000
6000
4000
2000
0
1960
1980
2000
2020
Total
Livestock
Wild

Figure S3: Uncertainty generated for the yearly herbivore metabolic consumption (a), biomass consumption in improved lands (b) and rangelands (c). Solid lines, dashed lines and shaded area represent respectively the mean, the minimum and maximum and the standard error of the yearly estimates.

Supplementary material S4. Results from the Generalized Additive Models

We used penalized regression splines to estimate the smooth term of the general additive model. Degrees of freedom were estimated to be optimal during the computation of the model using cross validation and a control parameter of maximum 4 threads. “Year” was modelled as a cubic spline.

Table S4: Selected results from GAM models. *Tot*, *Wild*, *Dom*, *Imp* and *Rang* stand for total, wild herbivore species, domestic herbivore species, improved lands, and rangelands. OVARI = sheep, BOSTAU = cattle, EQUFER = horse, CAPHIR = goats, RANTAR = reindeer, ANSALB = White fronted goose, ANSANS = Greylag goose, ANSBRA = pink footed goose, BRALEU = Barnacle goose, CYGCYG = whooper swan, LAGMUT = rock ptarmigan.

Variable	Edf	ref.df	Statistics (F)	p_value
**Total herbivore density**				
D_{tot}	8.986608	8.999936	3709.0846	< 0.001
**Species specific herbivore density**				
D_{OVIARI}	8.94	9.00	1602.70	< 0.001
D_{BOSTAR}	8.56	8.94	613.13	< 0.001
D_{EQUFER}	8.94	9.00	2225.27	< 0.001
D_{CAPHIR}	8.99	9.00	9560.57	< 0.001
D_{RANTAR}	7.80	7.98	1237.81	< 0.001
D_{ANSALB}	5.08	5.33	561.04	< 0.001
D_{ANSANS}	5.82	5.98	397.07	< 0.001
D_{ANSBRA}	7.95	8.01	1488.39	< 0.001
D_{BRALEU}	4.28	4.73	1031.97	< 0.001
D_{CYGCYG}	4.45	4.84	2100.00	< 0.001
D_{LAGMUT}	9.00	9.00	1788.97	< 0.001
**Total herbivore metabolic biomass**				
MB_{tot}				
**Group-specific herbivore metabolic biomass**				
MBW_{ild}	8.94	9.00	827.60	< 0.001
MB_{Dom}	4.52	4.92	25.01	< 0.001
**Total herbivore forage intake**				

DMI_{Tot_Imp}	8.93	9	1167	<0.001
DMI_{Tot_Rang}	8.93	9	1740	<0.001
**Group specific herbivore forage intake**				
DMI_{Wild_Imp}	6.54	6.81	428313	<0.001
DMI_{Wild_Rang}	6.23	6.48	24464	<0.001
DMI_{Dom_Imp}	8.93	9.00	988	<0.001
DMI_{Dom_Rang}	6.23	6.48	24464	<0.001

Supplementary material S5. Species-specific herbivore density trends

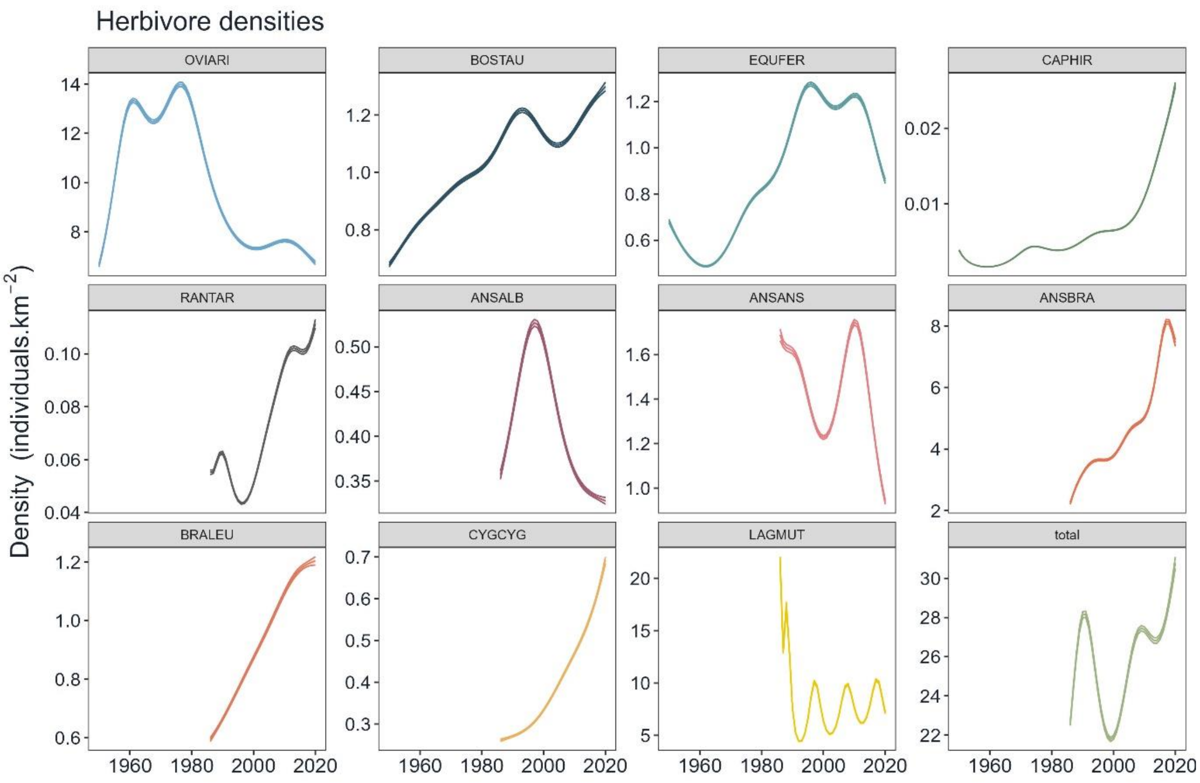

Figure S5: Species specific density trends in Iceland from fitted Generalized Additive Models with optimal degrees of freedom selected based on cross validation. Please note the different scales on the y-axes. OVARI = sheep, BOSTAU = cattle, EQUFER = horse, CAPHIR = goats, RANTAR = reindeer, ANSALB = White fronted goose, ANSANS = Greylag goose, ANSBRA = pink footed goose, BRALEU = Barnacle goose, CYGCYG = whooper swan, LAGMUT = rock ptarmigan. Blue gradients correspond to livestock species and red gradient wild species. Shaded areas around the lines represent the standard error of the estimates**.**

Supplementary material S6. Estimated net changes between 1986 and 2020

Table S6: Estimated net changes between 1986 and 2020 for herbivore densities, metabolic biomass, and forage intake.

Variable	Value in 1986	Value in 2020	Net changes*
**Total herbivore density**			
D_{tot}	22.6	30.8	36.4
**Species specific herbivore density**			
D_{OVIARI}	10.12	6.73	-33.70
D_{BOSTAU}	1.11	1.30	17.00
D_{EQUFER}	0.93	0.85	-8.43
D_{CAPHIR}	0.004	0.03	522.19
D_{RANTAR}	0.05	0.11	102.66
D_{ANSALB}	0.36	0.32	-8.02
D_{ANSANS}	1.67	0.94	-44.46
D_{ANSBRA}	0.94	7.44	231.47
D_{BRALEU}	0.59	1.20	101.94
D_{CYGCYG}	0.26	0.69	165.06
D_{LAGMUT}	21.70	7.17	-66.90
**Total herbivore metabolic biomass**			
MB_{tot}	144	126	-12.60
**Group-specific herbivore metabolic biomass**			
MBW_{ild}	18.1	17.5	-2.86
MB_{Dom}	135	108	-20.60
**Total herbivore forage intake**			
DMI_{Tot_Imp}	9537	8925	-6.42
DMI_{Tot_Rang}	6780	5745	-19.80
**Group specific herbivore forage intake**			
DMI_{Wild_Imp}	321	449	40.10
DMI_{Wild_Rang}	929	675	-27.4
DMI_{Dom_Imp}	9579	8724	-8.93
DMI_{Dom_Rang}	6711	5386	-19.8

*net changes of herbivore densities, metabolic biomass and forage intake were computed as 100 x (V_2 - V_1)/V_1, where V_1 is the estimated value for the earlier year and V2 is that for the last year.

Supplementary material S7. Averaged yearly net primary productivity within improved lands and rangelands in Iceland from 2000 to 2020

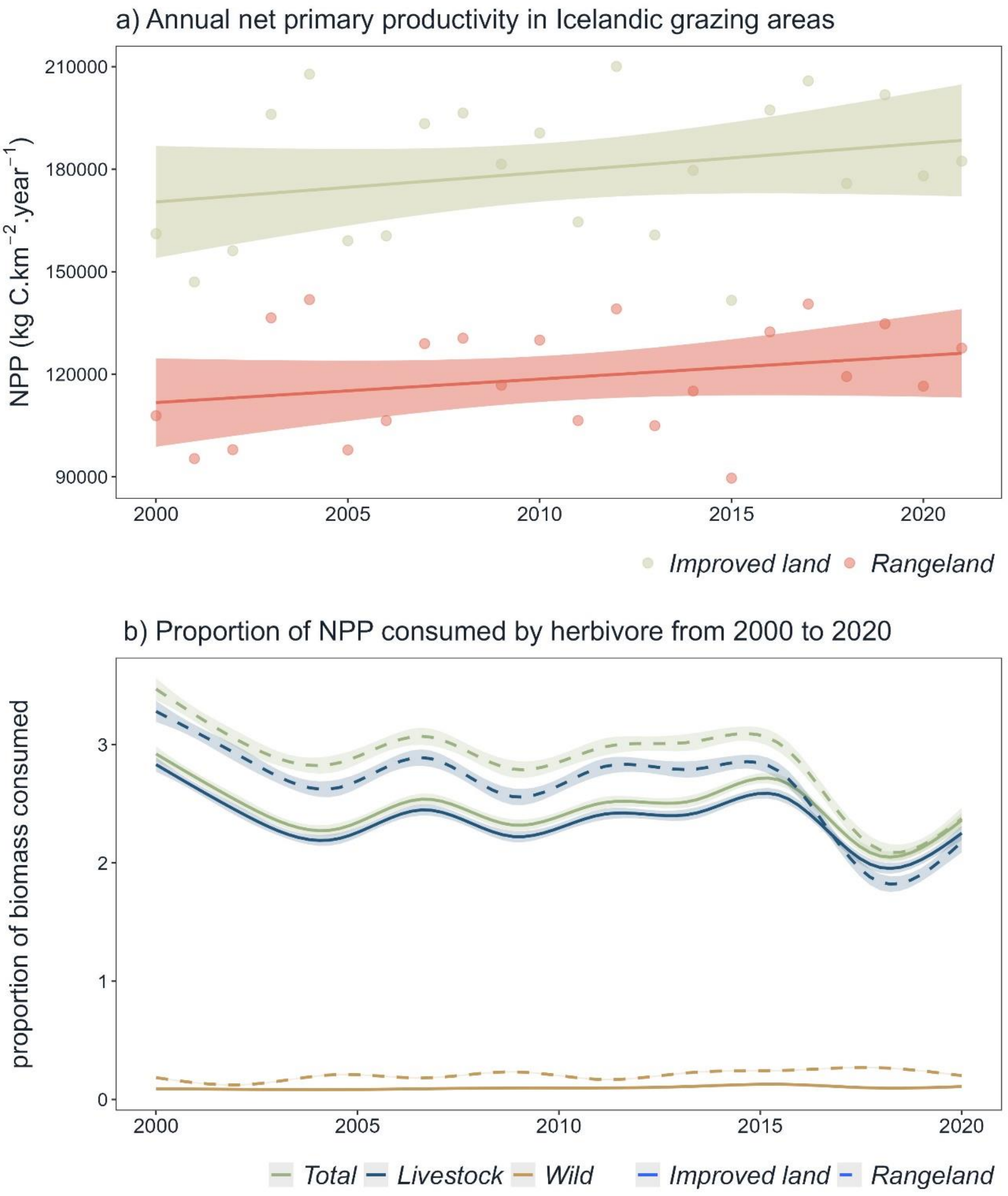

Figure S5: Averaged yearly net primary productivity (a) and proportion of plant biomass consumed by herbivores in grazing areas of Iceland (b) between 2000 and 2020. Solid lines correspond to improved lands and dashed lines to rangelands.
